# Dynamically correlated mutations drive human Influenza A evolution

**DOI:** 10.1038/srep02705

**Published:** 2013-09-19

**Authors:** F. Tria, S. Pompei, V. Loreto

**Affiliations:** 1 Institute for Scientific Interchange (ISI), Via Alassio 11C, 10126 Torino, Italy; 2 University of Turin, Physics Dept., Via Giuria 1, 10125 Torino, Italy; 3 Institute for Theoretical Physics, University of Cologne, Zülpicher Straβe 77, 50937 Köln, Germany; 4 Sapienza University of Rome, Physics Dept., Piazzale Aldo Moro 5, 00185 Roma, Italy

## Abstract

Human Influenza A virus undergoes recurrent changes in the hemagglutinin (HA) surface protein, primarily involved in the human antibody recognition. Relevant antigenic changes, enabling the virus to evade host immune response, have been recognized to occur in parallel to multiple mutations at antigenic sites in HA. Yet, the role of correlated mutations (epistasis) in driving the molecular evolution of the virus still represents a challenging puzzle. Further, though circulation at a global geographic level is key for the survival of Influenza A, its role in shaping the viral phylodynamics remains largely unexplored. Here we show, through a sequence based epidemiological model, that epistatic effects between amino acids substitutions, coupled with a reservoir that mimics worldwide circulating viruses, are key determinants that drive human Influenza A evolution. Our approach explains all the up-to-date observations characterizing the evolution of H3N2 subtype, including phylogenetic properties, nucleotide fixation patterns, and composition of antigenic clusters.

Influenza virus is estimated to infect yearly 5% to 20% of the United States population, with an average of ~40000 related deaths^1^,^2^. The major responsible of these high rates of morbidity and mortality, in the United States and worldwide, is the H3N2 subtype of Influenza A. The hemagglutinin (HA) surface protein of the virus has been the major focus of public health surveillance, due to its primary role in the interaction between the virus and the human immune system^3^. A crucial problem in the investigation and control of Influenza outbreaks is to unravel the complex interplay between the antigenic properties and the genetic profile of the virus. Each year sequences belonging to a single antigenic cluster are responsible for almost all the infections, and different antigenic clusters replace each other every 2–5 years^4^. This reflects in the peculiar structure of the phylogenetic tree, as inferred from the HA1 domain of the HA gene, characterized by a long trunk and short side branches representing closely related sequences that co-circulate every year^5^. This shape has been related to the continuous selective pressure that acts on the virus to evade hosts immunity^6^,^7^. Recently, transitions between antigenic clusters have been associated with multiple substitutions in the HA1 domain of hemagglutinin^8^,^9^. These observations strengthened previous results that highlighted how, although related, antigenic and genetic evolution do not follow the same patterns, antigenic evolution being more punctuated^10^. Further, genetic and antigenic distances between strains do not completely correlate: few amino acids substitutions can lead to strong differences in antigenic properties and conversely strains in the same antigenic cluster can exhibit high mutual genetic distance. Moreover, amino acid changes which seem to be relevant in the transition between two specific antigenic clusters, can exhibit a null antigenic effect when appearing in different sequences, so that changes in antigenic properties cannot simply be associated with key influential sites^10^.

Despite substantial results have been achieved in the effort of understanding the main mechanism driving the evolution of the Influenza A virus, fundamental questions such as how the extremely high mutation rate of the virus is compatible with its limited genetic diversity at each epidemic season, what are the determinants of its antigenic changes and what is the role of its global transmission dynamics in shaping its evolution remain largely unanswered^11^,^12^,^13^,^14^,^15^,^16^,^17^,^18^.

Early models of Influenza evolution lacked a clear distinction between genetic and antigenic distances and referred to isolated host populations. In that framework, multi-strains models^13^,^14^ were not able to reproduce the characteristic shape of the phylogenetic tree without invoking a temporary strain-transcendent immunity – after infection the host was hypothesized to acquire immunization against all the other strains for a period of some months. In particular, in the simplified model studied in^14^, lacking the host population structure considered in^13^, the temporary strain-transcendent immunity could not account alone for the comb-like shape of the Influenza A phylogenetic tree, and an a priory different infectivity of the different strains had to be considered as well. The interplay between a complex host population structure and the hypothesized generalized immunity thus remained a key question to be elucidated. A different perspective was later adopted in^15^, where static neutral clusters^19^,^20^ were adopted to introduce a genotype-phenotype mapping accounting for the difference in the genetic and antigenic evolution. This model was able to reproduce the main features of the Influenza A phylodynamics and to account for the variability of genetic similarity between strains in the same antigenic cluster. Yet, a self-consistent explanation of how jumps in clusters with substantial antigenically different properties are triggered was not proposed. In particular, relevant antigenic changes were explained by means of episodic strongly beneficial mutations. However, this mechanism has turned out to be inconsistent with the evolutionary pattern of this virus, where clonal interference coupled with a high and continuous rate of beneficial mutations have shown to play a relevant role in determining the selection of the strains^21^.

Here we show that a mechanism based on epistatic effects^22^, i.e., dynamically correlated mutations in antigenic sites, coupled with a reservoir that mimics worldwide circulating viruses, quantitatively accounts for shifts between antigenic clusters and allows to reconcile all the observations mentioned above, both experimental and theoretical, in a unique self-contained framework.

## Results

### The model

We consider a multi-strains stochastic model of virus transmission and evolution, where the interaction between host and virus is regulated by cross-immunity, depending on the antigenic properties of the viral sequence and on the host infection history. The epistatic mechanism we consider is such that jumps between different antigenic clusters are triggered whenever substitutions accumulate in groups of sites, which themselves depends on the evolutionary history of the virus, introducing in this way dynamically defined neutral clusters.

We define the antigenic distance between two strains as the maximal number of adjacent sites they differ on (we note that the adjacency of the sites is only a convenient, though general, way to model a group of suitable sites and it is not related to any biological insight). The antigenic space resulting from this definition of antigenic distance was studied in^23^, highlighting a non trivial structure of clusters of immunity. Here the ideas presented in^23^ are extended in a more realistic model of virus-host interaction. Two sequences elicit a complete cross-immunity against each other if their antigenic distance is lower that a fixed threshold *D*, otherwise a partial cross-immunity *σ* is considered. A sequence is assigned to an antigenic cluster whenever its antigenic distance from the cluster founder strain is lower than *D*. The cluster founder strain is the first emerged strain characterizing the novel antigenic cluster, i.e., exhibiting an antigenic distance higher than *D* from all the previous clusters' founder strains. The circulation dynamics at a global geographic level has been pointed out as a main mechanism through which the human Influenza A virus is sustained between seasonal epidemics^16^,^17^,^18^. This global transmission pattern is simplified in the model through the introduction of a reservoir that represents in a coarse-grained fashion the viral evolution outside the temperate region under consideration (see the Methods section for a detailed description of the evolutionary dynamics of the strains in the reservoir). In^15^ an immigration rate from an external reservoir was also introduced in order to avoid virus extinction but its implications for the model results were not fully investigated.

The epidemiological structure model is defined as follows: we consider a host (human) population of *N* individuals, each of which can host a viral strain. A viral strain is represented by a binary sequence of fixed length *L*. At each iteration step, the following processes are considered: with probability *Rdt* each infected individual tries to infect another one, randomly chosen in the population. The infection takes place with probability 1 − *σ* if the chosen individual is not infected itself and if none of the strains she was previously infected by elicits complete cross-immunity against the strain *i* carried by the infected individual. With probability *μdt* each viral sequence mutates a randomly chosen site; with probability *νdt* each infected individual recovers; with probability 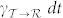 an individual is randomly chosen and, if infected, her viral sequence substitutes a sequence in the reservoir; with probability 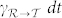 a sequence randomly extracted from the reservoir tries to infect a randomly chosen individual in the population (with the same mechanism discussed above). The time scale is set so to consider an infection period of a week (refer to the Methods section for further details).

### Outcomes

**Figure 1:**
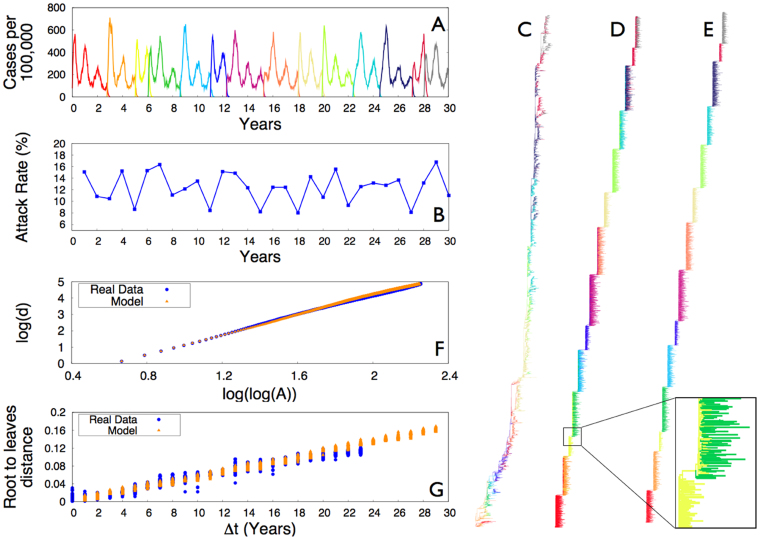
Infection pattern and phylogenetic properties. (A) Number of infected hosts as a function of time, as predicted by the model. Different colors correspond to different antigenic clusters. The average duration time of a single cluster is 2.5 years, with an excursion from 1 to 4 years. (B) Annual attack rate, i.e., the fraction of the population infected each year, as predicted by the model. (C) Phylogenetic tree as reconstructed from the HA1 sequence of 6859 viruses isolated between 1988 and 2011 (see Supplementary Information for details). (D) and (E) Phylogenetic trees as reconstructed from the model sequences, with respective assignment of sequences to clusters as described in the main text. In the zoomed area of (D) we focus on the superposition of two colors in the transition between the 3*rd* and the 4*th* cluster, due to a wrong attribution of sequences to antigenic clusters (see main text for discussion). (F) Mean depth of the phylogenetic trees in Panels C and D (or equivalently E) as a function of the total number of internal nodes and leaves *A*. Model's predictions are in striking agreement with real data (see details in the Supplementary Information). (G) Root to leaves distances vs. time (see text for details). The model predictions are in remarkable quantitative agreement with results from real data. The substitution rate of new alleles, as measured from the slope of a straight line fitting the plot, is *ρ_real_* = 5.29 · 10^−3^ substitutions/site/year. The parameters of the model corresponding to all the presented results are (refer to the main text for the definitions): *N* = 10^5^; *L* = 10^3^; *D* = 4; *σ* = 0.6 *μ* = 4.16 · 10^−3^ mutations/site/year; *ν* = 1; 
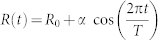
, with *R*_0_ = 2.0, *α* = 0.4, and *T* = 52; 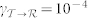 and 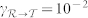; *dt* = 0.1.

The model reproduces the seasonal outbreaks of infection, with annual infection rate of 7% to 17%, and with antigenic clusters that replace each other every 1–4 years, with peaks of infection in correspondence of clusters transitions (Figs. 1, A–B)^24^. The assumptions of our model are validated by means of a thorough comparison between the model results and measures performed on HA1 sequences of human H3N2^25^. We restrict the analysis to nucleotide sequences isolated from 1988 to 2011, for which a substantially larger number of yearly isolates is available with respect to previous years, and more attention has been paid in avoiding sampling biases^26^. Strains are assigned to clusters of immunity according to the vaccine composition recommendation of their year of isolation^27^. This definition differs from the antigenic cluster classification based on the hemagglutination inhibition (HI) assay titer, in particular updates of the vaccine strains was often needed more than once within a single antigenic cluster as defined in^10^. In Fig. 1 (C,D,E) we report respectively the phylogenetic trees as reconstructed from the Influenza sequences (C) and from the sequences generated by the model (D,E), where different antigenic clusters are shown in different colors. It is worth observing that the criterion based on vaccine recommendation can result in a wrong attribution of the antigenic clusters for sequences isolated in years where clusters transitions took place, reflecting in the presence of two colors on the phylogenetic tree's branches of those years. This artifact can however be revealed by the model. In the tree in Fig. 1D, sequences generated by the model are associated to the antigenic cluster responsible for pandemics in the year of their sampling. With this assignment two different colors can coexist in the subtrees corresponding to cluster transitions (in the zoomed area of Fig. 1D, for example, we focus on the transition between the 3*rd* and the 4*th* cluster). In Fig. 1E we show the same phylogenetic tree, but where strains are associated with their actual cluster of immunity, such that the superposition of two colors in the same year disappears. The phylogenetic tree reconstructed from the model's sequences appears less structured within each year with respect to the one reconstructed from the Influenza sequences data, due to the oversimplifying assumption in our modeling scheme of not considering several geographic regions. However, its global structure features an extremely good agreement with the Influenza tree. In order to show that, we focus on a measure of imbalance that has been shown^28^ to efficiently discriminate between different evolutionary processes of RNA viruses. Fig. 1F displays the mean depth of the phylogenetic trees shown in panels C and D (or equivalently E) as a function of the total number of internal nodes and leaves *A* (*A* = 2*n* − 1 in a rooted binary tree with *n* leaves) of subtrees sampled from the complete one. The mean depth is defined^28^ as 
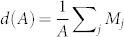
, where *M_j_* is the topological distance of the node *j^th^* (leaf or internal) from the root. This measure shows the remarkable agreement between the model predictions and the real data. Finally, Fig. 1G displays the root to leaves distances vs. time, i.e. the percentage of genomic substitutions of strains sampled over time from the founder strain, as measured from the phylogenetic trees in panels C and D (or equivalently E). The substitution rate per site predicted by the model is compared with the H3N2 nucleotide substitution rate in the HA1 domain, showing again a remarkable quantitative agreement.

**Figure 2:**
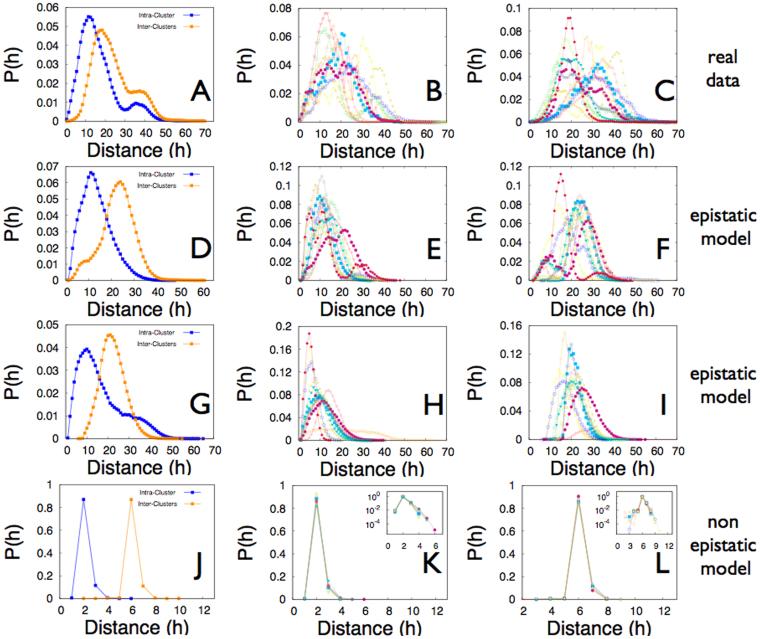
Properties of the antigenic clusters. (A) Distributions of the Hamming distances *h* between any possible pairs of strains assigned to the same antigenic cluster (Intra-cluster), or any possible pairs of strains assigned to two consecutive clusters (Inter-clusters), as measured from the 987 nucleotides of the HA1 domain of the haemagglutinin gene (HA) of the 6859 viruses isolated between 1988 and 2011. The sequences' antigenic clusters are defined according to the vaccine composition recommendation for the correspondent year, as discussed in the main text. The plot is an average over all the available antigenic clusters. (B) Intra-clusters distributions for the same data as in A, separately reported for each antigenic cluster. (C) Inter-clusters distributions for the same data as in A, separately reported for each pair of consecutive antigenic clusters. (D) Same measures as in (A), for the strains produced by the epistatic model. Here the strains are associated to the antigenic cluster responsible for pandemics in their year of sampling. Panels (E) and (F) are the equivalent of panels B and C, for the model data analyzed in panels D. (G), (H), (I) Same data as (D,E,F) but with the strains associated with their actual antigenic cluster. Panels (J), (K), (L) Same data as (G,H,I) but for the non-epistatic (NE) model.

**Figure 3:**
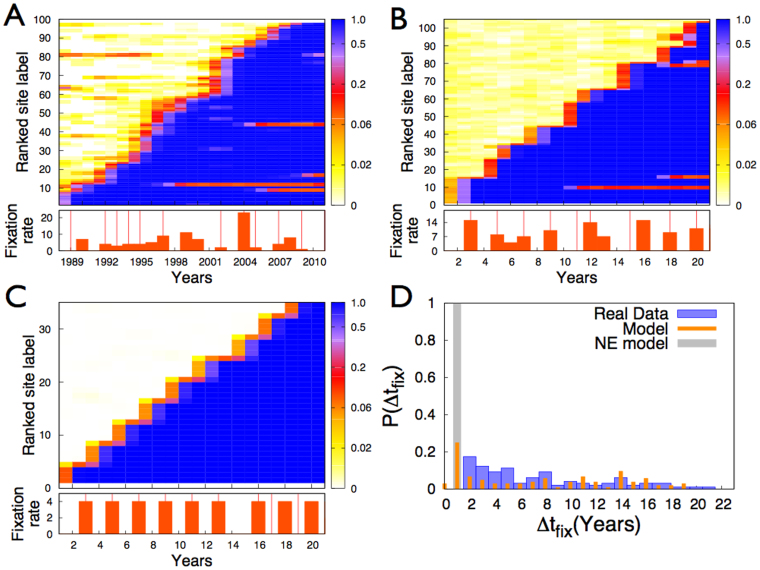
Patterns of fixations of nucleotide substitutions. (A) Temporal map of new alleles frequencies for substitutions that undergo fixation, for the 987 nucleotides of the HA1 region of the haemagglutinin gene (HA) of 6859 viruses isolated between 1988 and 2011. (B) Same as A for the sequences generated by the model with epistasis. (C) Same as A for the sequences generated by the NE model. The temporal maps are constructed as follows: in the y-axes the label of each site corresponds to its rank with respect to the year of fixation, a lower number corresponding to an earlier fixation. With a color code is then shown, for each site of the detected substitutions, the allele frequency of the new allele that will undergo fixation, at each considered year (x-axes). The graphs below panels A, B and C report the number of substitutions fixed per year, both for real data and data model. Here vertical red lines mark transitions between antigenic clusters. (D) Histograms of the fixation times Δ*t_fix_* for substitutions. Here the fixation time is defined as the timespan between the first occurrence of a substitution (defined as present in at least 1% of the circulating strains) to its fixation (defined as present in 95% of the circulating strains).

We now turn to a deeper investigation of the antigenic evolution as predicted by the model, and as measured from the Influenza sequences. We find that the model is able to reproduce both the genetic variability within and between consecutive antigenic clusters (Fig. 2), and the pattern of sites substitutions (Fig. 3), in quantitative agreement with the measures on the Influenza sequences data. In particular, the genetic distances between sequences belonging to the same antigenic cluster and between sequences belonging to two consecutive clusters, well reflect genetic distances as measured from the HA1 nucleotidic sequences of the H3N2 virus (Fig. 2, A–I). We note both in panels D and G a significant overlap between the Intra-cluster and Inter-clusters distributions, as observed in real data (Panel A). A surprising quantitative agreement is also recovered for the mean values of the distributions: 〈*h*〉 = 18.32, 〈*h*〉 = 13.56, 〈*h*〉 = 16.66 for the Intra-cluster distributions and 〈*h*〉 = 24.93, 〈*h*〉 = 22.66, 〈*h*〉 = 22.22 for the Inter-clusters distributions, respectively in panels A, D and G. Further, a great variability in the Intra-cluster distributions related to different antigenic clusters, and in the Inter-clusters distributions related to different consecutive antigenic clusters is observed in the model results (Panels E and H and panels F and I respectively), as well as in real data (Panels B and C respectively). In Fig. 3 we explicitly explore the pattern of nucleotide substitution in the sequences generated by the model and in the Influenza sequences. We observe a striking agreement between the Influenza data (A) and the model predictions (B) as for the patterns through which alleles get fixed in the population and, quite surprisingly, as for the total number of substitutions observed in the same time lapse. In particular, both in Influenza data and in the model, multiple fixations are observed in correspondence to antigenic clusters transitions, with a high variability in the number of simultaneous fixations. The latency time since the first appearance of a new allele to its fixation exhibits a large distribution both in the Influenza data and in the model data (Fig. 3C), as already observed for amino acids substitutions in^8^.

### The role of epistasis

In order to shed light on the crucial role epistasis is playing in shaping the evolution of the Influenza virus, we further consider a version of the model where epistatic effects are removed, by setting the antigenic distance as proportional to the genetic one. This non-epistatic (NE) model is thus structured precisely as the one with epistasis, with the only change in the definition of antigenic distance and consequently of the antigenic clusters. In the NE model the antigenic distance between two sequences is simply defined as their genetic distance *h*, i.e. the Hamming distance, the number of homologous sites at which two strains differ. Remarkably, the NE model is still able to quantitatively account for the limited genetic diversity of the hemagglutinin sequences at each epidemic season as well as for the continuous replacement of antigenic clusters (refer for this to the Supplementary Information). These findings suggest that the above mentioned properties are mainly related to the global circulation pattern of the virus, along with its short infection period. To our knowledge, this is the first time that this implication has been highlighted.

We studied the NE model both for the same value of the cross-immunity threshold *D* as considered in the epistatic model, and for a sensibly higher value of *D* (refer for this to the Supplementary Information), such that a realistic value for the substitution rate is recovered. Without epistatic effects in point mutations it is not possible to reproduce the genetic variability within and between antigenic clusters, nor the amino acids substitutions patterns as experimentally observed. In particular, in the NE model, with any value of *D*, the distributions of Hamming distances of the strains inside the same antigenic cluster and across two consecutive clusters do not feature any overlap (Fig. 2J and Supplementary Information), as observed instead in real data and in the model with epistasis. Further, both distributions do not exhibit any variability between different clusters (see Figs. 2, K and L and Supplementary Information), again marking a difference with respect to the measures performed on real data and to the results of the model with epistasis. Moreover, the NE model features patterns of fixation of sites mutation (Fig. 3, C and D and Supplementary Information) significantly different from those observed in the Influenza data. The NE model cannot thus capture the richness of the real data on human Influenza A. The number of sites that get fixed in correspondence of clusters transitions exhibits a very poor variability (Fig. 3,C and Supplementary Information) and we do not observe single point mutations that persist in a small fraction of the population for many years before getting fixed (Fig. 3, C and D). The NE model predicts in fact a fixation time of 1 year for all the nucleotide mutations (Fig. 3D and Supplementary Information).

## Discussion

In summary, we have introduced a modelling framework to investigate the processes through which epistasis, i.e., a departure from independence of the effects of mutations in different genetic loci, can affect the phylodynamics of Influenza A virus. We coupled a multi-strains model of virus transmission and evolution with a dynamics of *immigration* and *emigration* from and towards a reservoir that mimics the global transmission dynamics of the virus. By specifying the genotype-phenotype mapping (the phenotype being in this context the antigenic properties of a virus), epistasis plays a crucial role on the evolutionary dynamics of the virus. Overall, we find that the interplay between dynamically correlated mutations in the genomic region under selective pressure by the host immune system, and a transmission dynamics that ensures the virus survival through circulation patterns at a global scale, is able to explain the phylodynamics of the human Influenza A as well as its antigenic evolution. The global transmission dynamics can reproduce, even without epistatic effects, the limited genetic diversity of the hemagglutinin sequences at each epidemic season and the continuous replacement of antigenic clusters. However, the substitution rate predicted by the model without epistasis features realistic values only for values of the cross-immunity threshold as high as *D* ~ 15 (refer to Supplementary Information). Most importantly, it is only when epistatic effects are taken into account that the genetic variability within and between consecutive antigenic clusters can be reproduced in a quantitative way (Fig. 2 and Supplementary Information). Further, epistatic effects are essential to explain the pattern of fixations in antigenic sites (Fig. 3 and Supplementary Information), as observed in the HA1 nucleotidic sequences of the H3N2 virus. We think these results, by shedding light on the implications of both the global transmission dynamics and of epistatic interactions, could pave the way to a more thorough comprehension and control of the determinants of the Influenza A virus evolution. In addition, the enhanced understanding of the complex interplay between the antigenic properties and the genetic profile of the virus can trigger progress both for worldwide spreading models^29^ and prevention strategies.

## Methods

### Definition of the antigenic distance and of the antigenic clusters

A viral strain is modeled as a binary sequence *s* of length *L* and the *genetic distance h* between two strains is defined as the number of homologous sites they differ on (*Hamming distance*). In order to take into account epistatic effects, we define the *antigenic distance E* between two strains as the maximum number of adjacent sites they differ on. For instance, the two sequences: 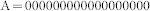
and 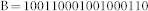
have genetic distance *h*(*A*,*B*) = 7 and antigenic distance *E*(*A*,*B*) = 2. We consider the cross-immunity elicited by a strain against another (and viceversa) as complete if the antigenic distance between the two strains is not greater than a fixed threshold *D*, otherwise we consider a partial cross-immunity: the probability that an host previously infected with one of the two strains can be later infected by the other is *σ* < 1.

In order to identify antigenic clusters (or clusters of immunity), we define the progenitor or founder strain of the *i*-th cluster as the first sequence, say *s_i_*, appearing either in the reservoir or in the temperate region, that evades the complete immunity of all the previous *i* − 1 clusters' founders (the founder strain of the first cluster is the first strain appearing in the population). A strain *s* is associated to a cluster *i* if *E*(*s*, *s_i_*) ≤ *D*. This definition is not univocal, since a strain can satisfy the inequality for more than one cluster: in this case it is associated to the most recent one.

### Dynamics in the temperate region 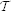


We consider a population of *N* individuals, each of whom can host a viral strain. Each individual can be in one of the following two states:

**I:** *Infected*, by a unique strain *s*;

**S:** *Susceptible* (if not infected) to the infection by suitable strains of the virus, depending on its acquired immunity.

The immunity acquired by any individual *i* is determined by the set of strains 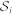 she has been infected by in the past. A susceptible individual *i* has a complete immunity against a strain *s* (cannot be infected by *s*) if the set 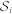 contains at least a strain *s_k_* such that *E*(*s_k_*, *s*) ≤ *D*. Otherwise, she can be infected by *s* with a probability *σ* < 1.

At each time step an infected individual *k* is chosen randomly. One first checks for possible mutations of the viral strain: with probability *μ dt* the strain mutates a random site. Let us call *s* the resulting strain. Further, with a probability *R*(*t*) *dt* one of the *N* − 1 remaining individuals is picked up randomly and if she is susceptible and her immune memory does not elicit complete immunity, she becomes infected by the strain *s* with probability 1 − *σ*. Finally, with probability *νdt* the individual *k* is recovered and the strain *s* is added to her immune memory set 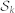.

### Dynamics in the virus reservoir 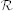


In order to simulate the global circulation dynamics, we consider a reservoir of *N* strains, which represents, in a coarse-grained fashion, the viral evolution outside the temperate region under consideration. The dynamics of the virus is regulated by rounds of mutation and selection, through a genetic algorithm. To each strain *i* is assigned a fitness, which is time-dependent and depends on its cluster of immunity *k*, defined as: 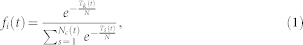
where *N_c_*(*t*) is the total number of clusters of immunity at time *t* and *T_k_*(*t*) is the number of strains associated to the cluster *k* from its appearance to time *t*.

The fitness in (1) is such that newly appeared clusters have a higher probability of survival. This mimics the dynamics of the strains in a population of individuals.

At each time step, a virus is picked up randomly in the population and with probability *μ* *dt* it undergoes a mutation. The selection of the strains occurs every 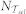 time steps. During the selection, strains are sampled and copied in the next generation, with a probability proportional to their fitness (1).

### Interaction between 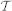 and 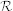


The two regions 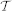 and 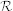 can exchange viruses with emigration and immigration events. At each time step, a virus, say *s_imm_*, is randomly chosen from the reservoir, and moves to the region 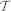 (immigration) with a probability 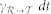. If the immigration event takes place, with probability *R*(*t*) *dt* an individual in the temperate region is picked up randomly and if she is susceptible and her immune memory does not elicit complete immunity against *s_imm_*, she becomes infected by the strain *s_imm_* with probability 1 − *σ*. An emigration event occurs at each time step with probability 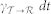: an individual in 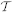 is picked up randomly and, if it is infected, say by the strain *s_emi_*, the virus *s_emi_* enters in the reservoir, replacing one of the existing strains.

### Parameters selection

We chose the parameters values in agreement with realistic estimates, whenever available, or such that to reproduce realistic estimates of related quantities. For more general choices of the parameters set we shall always discuss the robustness of the model with respect to their changes (refer for this to the Supplementary Information).

#### Time scales

A week is chosen to be the unit of time, by setting the recovery time *ν* = 1. Correspondingly, we set the selection time in the reservoir *τ_sel_* = 1, so that selection in the reservoir occurs at the same time scale of the average duration of an infection. The elementary time step is set to *dt* = 0.1, i.e., about 17 h.

#### Sequence length

We consider binary sequences of length *L* = 1000, in accordance with the 987 nucleotides of the HA1 domain of the haemagglutinin (HA) segment in the human influenza virus.

#### Basic reproductive number

The mean number of infection attempts *R*_0_ caused by an infected individual follows a sinusoidal behavior, reproducing seasonal fluctuations, of the form: 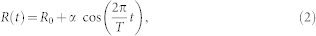
where *R*_0_ is usually called the basic reproductive number and we set an oscillation period of one year, i.e. *T* = 52 in units of weeks. We set *R*_0_ = 2^30^,^31^ and *α* = 0.4^31^,^32^. In the Supplementary Information, we will show how the main observables of the model depend on *R*_0_ and *α* over a wide range of realistic values.

#### Cross-immunity

Cross-immunity elicited by a strain against another is set to be total (*σ* = 1) if the two strains have antigenic distance lower or equal to *D*, otherwise *σ* = 0.6^33^,^34^,^35^. In the Supplementary Information, we will show how the main observables of the model depend on the considered value of the *σ* parameter.

#### Complete cross-immunity threshold

The threshold *D* is set to *D* = 4 in all the results in the main text. We will show how the results depend on this threshold in the Supplementary Information.

#### Emigration and immigration rates

The emigration and immigration rates are set respectively equal to 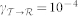 and 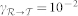, fulfilling the inequality 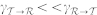
16,17,18. Again, a discussion on the dependence of the model results on these two rates is given in the Supplementary Information.

#### Population size and mutation rate

Finally, we set a population size of *N* = 100000 and a mutation rate *μ* = 4.16·10^−3^ per site per year. In the Supplementary Information we will also discuss the scaling properties of the model with respect to these two parameters.

## Author Contributions

F.T., S.P. and V.L. designed the experiments, analyzed the data and wrote the paper.

## Supplementary Material

Supplementary Information Supplementary Information
