# Supplementary material for: Dynamically correlated mutations drive human Influenza A evolution
Source: Sci Rep. 2013 Sep 19;3:2705. doi: 10.1038/srep02705 (PMC3776956; doi:10.1038/srep02705)
Supplement: Supplementary Information [file srep02705-s1.pdf]

Dynamically correlated mutations drive human Influenza A evolution

Supplementary Information

F. Tria[1,*], S. Pompei[2,1,3], V. Loreto[4,1]

[1]Institute for Scientific Interchange (ISI), Via Alassio 11C, 10126 Torino, Italy
[2]University of Turin, Physics Dept., Via Giuria 1, 10125 Torino, Italy
[3]Institute for Theoretical Physics, University of Cologne, Zülpicher Straße 77, 50937 Köln, Germany
[4]Sapienza University of Rome, Physics Dept., Piazzale Aldo Moro 5, 00185 Roma, Italy

1 Phylogenetic properties of the modified model without epistasis (NE model)

In this section we present the results obtained for the model without epistasis (the NE model introduced in the main text). The model is structured precisely as the one with epistasis, the only difference being the definition of antigenic distance and consequently of the antigenic clusters. Here the antigenic distance between two sequences is simply defined as their genetic (Hamming) distance h. All the parameters are set as discussed for the main model, but the mutation rate μ. This is set in order to recover a realistic rate of clusters replacement. As discussed in the main text, the infection pattern and the phylogenetic properties of the Influenza A are well reproduced by this model as well (Fig. S1, A–C, and Fig. S2, A–C). On the other hand, with this value of μ, the substitution rate as measured on the phylogenetic tree is well below a realistic value if we set $D = 4$ as in the main text (Fig. S1, D), while it reproduces the human Influenza A substitution rate as measured from real data (as discussed in the main text), when D is set to a sensibly higher value, namely $D = 15$ (Fig. S2, D).

However, with any value of D, the non epistatic model is not able to reproduce a realistic genetic variability for the antigenic clusters, nor a realistic fixation

pattern of sites mutations (refer for this to the main text for results with $D = 4$, and to Figs. S7, S8 and S9 for results with $D = 15$).

2 Dependence of the models, both with and without epistasis, on the parameters values

2.1 Dependence on the threshold D defining the antigenic clusters

In this section we discuss how the ensemble of all the results presented in the main text depends on the value of the antigenic threshold D. We consider in particular $D = 2$ and $D = 3$, both for the models with and without epistasis, and the additional value $D = 15$ for the model without epistasis. We show that, provided one chooses a suitable value for the mutation rate μ, realistic patterns of infection and of clusters replacement can be reproduced (Fig. S3), as well as a realistic levels of imbalance of the phylogenetic trees (Fig. S4). However, as in the case of the model without epistasis with $D = 4$, the resulting substitution rates remains well below the actual empirical values in both model when $D < 4$ (Fig. S5).

In Fig. S6 and S7 we report the genetic variability of the sequences within antigenic clusters and between sequences belonging to consecutive antigenic clusters, for the model with epistasis and for the model without epistasis respectively. We report here for comparison also the results for $D = 4$ (already shown in the main text for both models with and without epistasis). The left column of each figure reports the overall intra-cluster and inter-clusters distributions, $P_{inter}(h)$ and $P_{intra}(h)$ respectively, of the hamming distances between couples of strains, while the center and right column show respectively the intra-cluster and inter-clusters distributions within each cluster and between pairs of consecutive clusters.

In the model with epistasis we observe a remarkable overlap between intra-cluster and inter-clusters distributions already for $D = 3$, becoming more evident for $D = 4$. The mean values of the distributions $P_{intra}(h)$ and $P_{inter}(h)$ read: $< h >_{intra} \approx 2$ and $< h >_{inter} \approx 4$ for $D = 2$; $< h >_{intra} \approx 4$ and $< h >_{inter} \approx 9$ for $D = 3$; $< h >_{intra} \approx 17$ and $< h >_{inter} \approx 22$ for $D = 4$. The values for $D = 4$ are remarkably close to the values measured for the corresponding Influenza A distributions (shown in the main text), which read $< h >_{intra} \approx 18$ and $< h >_{inter} \approx 25$.

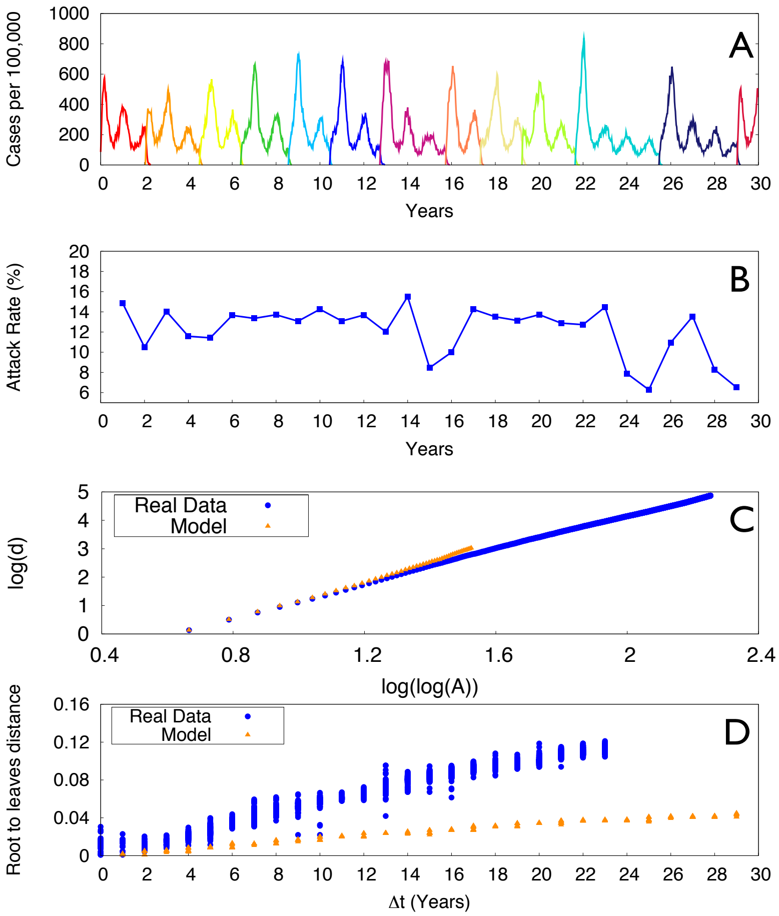

Figure S1: Infection pattern and phylogenetic properties for the NE model. The values of the model parameters used here are: $N = 100000$; $L = 1000$; $D = 4$; $\sigma = 0.6$ $\mu = 5.772 \cdot 10^{-5}$ mutations/site/year; $\nu = 1$; $R(t) = R_0 + \alpha \cos\left(\frac{2\pi t}{T}\right)$, with $R_0 = 2.0$, $\alpha = 0.4$, and $T = 52$; $\gamma_{\mathcal{T}\to\mathcal{R}} = 10^{-4}$ and $\gamma_{\mathcal{R}\to\mathcal{T}} = 10^{-2}$. (**A**) Fraction of infected hosts as a function of the time. Different colors correspond to different antigenic clusters, higher peaks of infections marking cluster jumps. The average duration time of a single cluster is 2.5 years, with an excursion from 1 to 4 years. (**B**) Annual attack rate infection rate as predicted by the model. (**C**) Mean depth of the phylogenetic tree as predicted by the model without epistasis. For further details we refer to the Section 3.1). (**D**) Percentage of genomic substitutions of strains sampled over time from the founder strain (the root of the tree), as measured from the phylogenetic tree. The substitution rate of new alleles, as measured from the slope of a straight line fitting the plot, is $\rho = 1.6 \cdot 10^{-3}$ substitutions/site/year, significantly lower than the value measured for the Influenza A virus (see Fig. 1 of the main text for comparison).

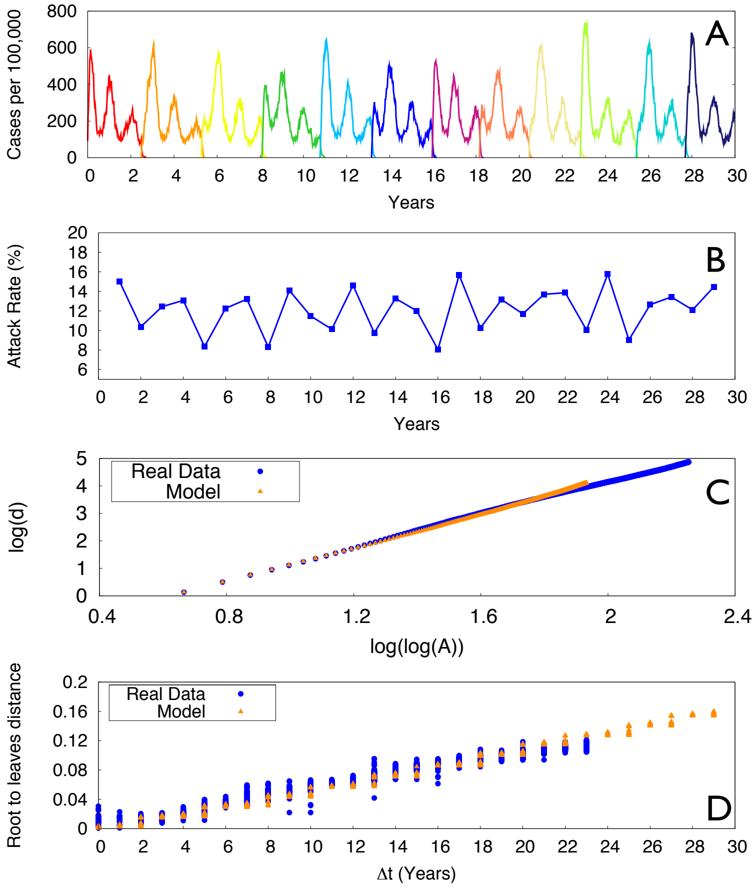

Figure S2: Infection pattern and phylogenetic properties for the NE model. The values of the model parameters used here are: $N = 100000$; $L = 1000$; $D = 15$; $\sigma = 0.6$ $\mu = 1.56 \cdot 10^{-3}$ mutations/site/year; $\nu = 1$; $R(t) = R_0 + \alpha \cos\left(\frac{2\pi t}{T}\right)$, with $R_0 = 2.0$, $\alpha = 0.4$, and $T = 52$; $\gamma_{\mathcal{T}\to\mathcal{R}} = 10^{-4}$ and $\gamma_{\mathcal{R}\to\mathcal{T}} = 10^{-2}$. (**A**) Fraction of infected hosts as a function of the time. Different colors correspond to different antigenic clusters, higher peaks of infections marking cluster jumps. The average duration time of a single cluster is 2.42 years, with an excursion from 1 to 4 years. (**B**) Annual attack rate infection rate as predicted by the model. (**C**) Mean depth of the phylogenetic tree as predicted by the model without epistasis. For further details we refer to the Section 3.1). (**D**) Percentage of genomic substitutions of strains sampled over time from the founder strain (the root of the tree), as measured from the phylogenetic tree, for the model and for the influenza A data.

In the model without epistasis, for all the four different values of the threshold $D = 2, 3, 4, 15$ the overlap between the intra-cluster and inter-clusters distributions is almost absent. Further, the mean values $< h >_{intra}$ and $< h >_{inter}$ are far from the corresponding values for Influenza A. In particular, for the values $D = 2, 3, 4$, the intra-cluster distributions are always peaked in $h = 2$, while the inter-clusters distributions have a maximum peak in $h = 6$ for $D = 4$. For $D = 15$, the mean values of the distributions $P_{intra}(h)$ and $P_{inter}(h)$ read $< h >_{intra} \approx 6$ and $< h >_{inter} \approx 20.5$.

In Fig. S8 we report the temporal maps of the frequencies of new alleles appearing in the population for the models with and without epistasis and for $D = 2$ and $D = 3$, for the model with epistasis and $D = 4$ (same picture of the main text) and for the model without epistasis and $D = 15$. The detected substitutions are grouped in the y axis according to their year of fixation. In order to quantify the annual amount of newly fixed mutations, we also show in the banners the fixation rates, i.e., the variation of the total number of fixed sites with respect to the previous year. The left column shows the results for the model with epistasis, for $D = 2$ (top), $D = 3$ (center), and $D = 4$ (bottom), while the right column shows the results for the model without epistasis, for $D = 2$ (top), $D = 3$ (center), and $D = 15$ (bottom). In all cases fixations of mutations take place after the appearance of a new cluster of immunity. In the model without epistasis, the fixation rate always reaches a maximum equal to D, since D mutations are sufficient for the appearance of a new antigenic cluster. Further, the appearance of every antigenic cluster is marked by roughly the fixation of D mutations. On the contrary, in the model with epistasis the number of fixations after the appearance of a new cluster features a greater variability already for $D = 3$, with a pattern similar to the one observed in the Influenza data. A quantitative agreement with Influenza data is recovered when $D = 4$ (see main text for comparison).

In Fig. S9 we report the histograms of the fixation times Δt_{fix} for substitutions, i.e., the timespan between the first occurrence of a substitution to its fixation in the population. We consider here two different values for the sequences fraction in which a substitution should appear to be considered present in the population (namely in the 1% of the circulating strains as in the main text, and in the 0.5% of the circulating strains). We compare results for both the models with and without epistasis and real data outcomes. We here consider the values $D = 4$ for the model with epistasis and $D = 15$ for the NE model, expanding the analysis already performed in the main text. While a remarkable agreement with real data is featured by the epistatic model, the NE model fails in reproducing the variability in the fixation times as observed for the human Influenza A.

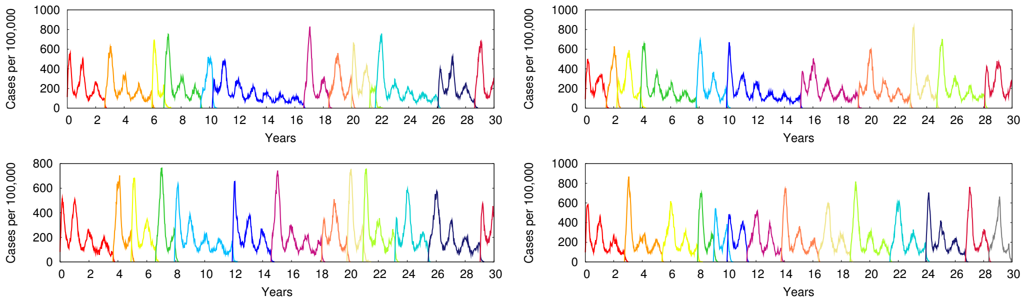

Figure S3: Infection patterns. Fraction of infected hosts as a function of time, different antigenic clusters being depicted in different colors. We report examples of a realistic pattern, obtained both with the model with epistasis (Left column) and with the model without epistasis (Right column). The first raw corresponds to results for $D = 2$ and the second raw corresponds to results for $D = 3$. In all the simulations, we set the parameters values: $N = 100000$; $L = 1000$; $D = 4$; $\sigma = 0.6$; $\nu = 1$; $R(t) = R_0 + \alpha \cos\left(\frac{2\pi t}{T}\right)$, with $R_0 = 2.0$, $\alpha = 0.4$, and $T = 52$; $\gamma_{\mathcal{T}\rightarrow\mathcal{R}} = 10^{-4}$ and $\gamma_{\mathcal{R}\rightarrow\mathcal{T}} = 10^{-2}$. For the model with epistasis and $D = 2$ (top left) we show results for $\mu = 3.64 * 10^{-5}$ mutations/site/year, with a resulting $\Delta t_{clusters} = 2.42$ years and $\langle IPR \rangle = 1.01$ (See Section 2.2 for the definitions of $\Delta t_{clusters}$ and of the Inverse Participation Ratio, $\langle IPR \rangle$). For the model without epistasis and $D = 2$ (top right) we show results for $\mu = 1.56 * 10^{-6}$ mutations/site/year, with a resulting $\Delta t_{clusters} = 2.89$ years and $\langle IPR \rangle = 1.01$; for the model with epistasis and $D = 3$ (bottom left) we show results for $\mu = 7.8 * 10^{-4}$ mutations/site/year, with a resulting $\Delta t_{clusters} = 2.43$ years and $\langle IPR \rangle = 1.01$; for the model without epistasis and $D = 3$ (bottom right) we show results for $\mu = 1.456 * 10^{-5}$ mutations/site/year, with a resulting $\Delta t_{clusters} = 2.14$ years and $\langle IPR \rangle = 1.02$. In all the cases a realistic infection pattern and antigenic clusters replacement can be found, for a suitable mutation rate μ.

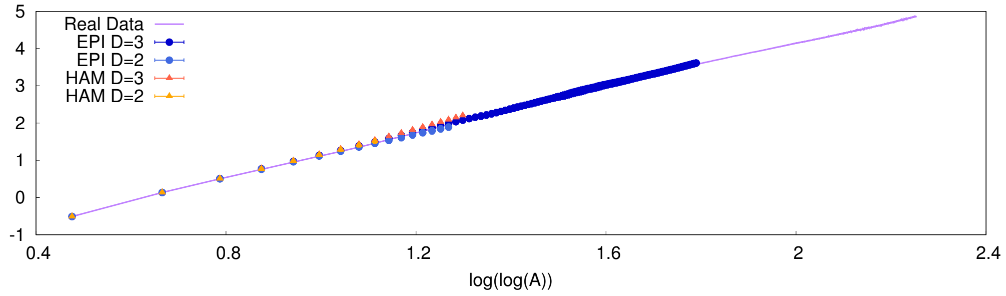

Figure S4: Mean depth d of the phylogenetic tree (refer to Section 3.1 for the definition). Values of the model parameters as in Fig. S3. For all the cases considered the level of imbalance of the phylogenetic tree turns out to be in very good agreement with real data.

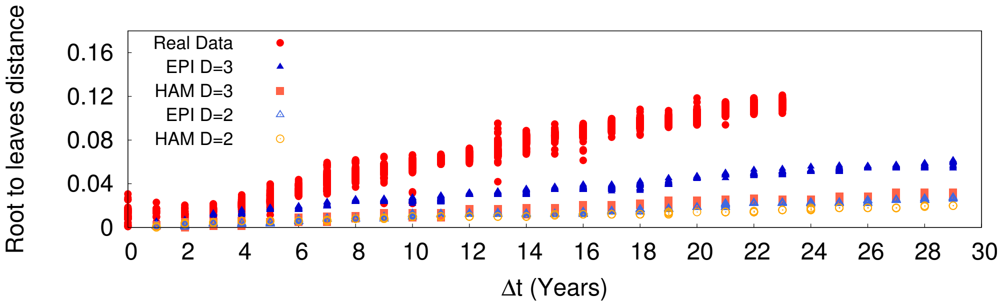

Figure S5: Substitution rates. Percentage of genomic substitutions of strains sampled over time from the founder strain, as measured on the phylogenetic tree. Different data sets corresponds to real data, the model with and without epistasis (NE and EPI respectively), for different values of the threshold ($D = 2, 3$. The substitution rates of new alleles, as measured from the slope of a straight line fitting the plot, are respectively: $\rho_{EPI}^{D=2} = 9.1 \cdot 10^{-4}$ substitutions/site/year for the model with epistasis and $D = 2$; $\rho_{EPI}^{D=3} = 2.0 \cdot 10^{-3}$ substitutions/site/year for the model with epistasis and $D = 3$; $\rho_{NE}^{D=2} = 7.1 \cdot 10^{-4}$ substitutions/site/year for the model without epistasis and $D = 2$; and $\rho_{NE}^{D=3} = 1.1 \cdot 10^{-3}$ substitutions/site/year for the model without epistasis and $D = 3$. Here the resulting values of the substitution rates are always significantly lower than the estimated values for Influenza A. Values of the model parameters are set as in Fig. S3.

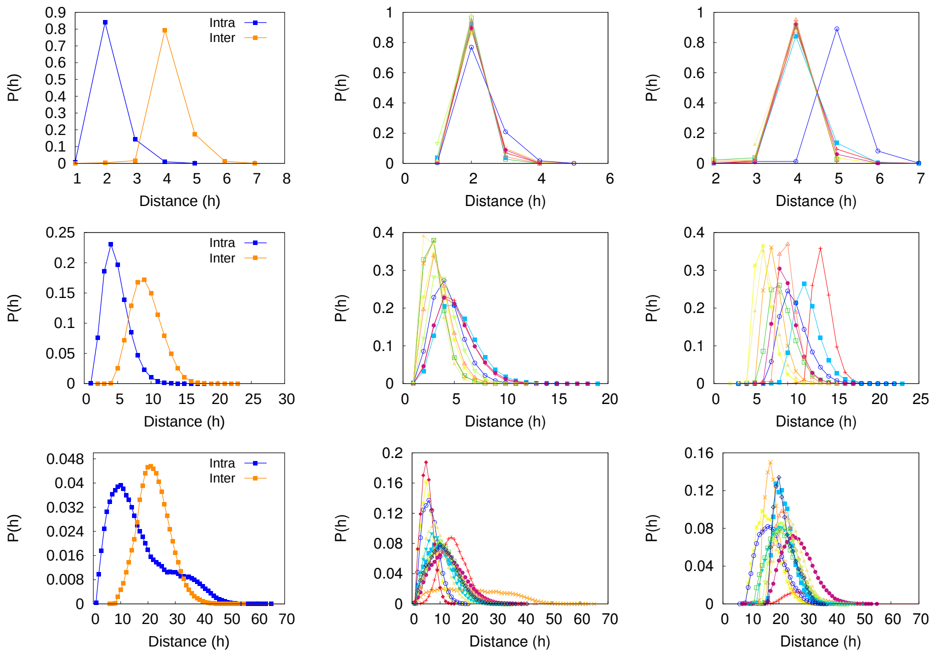

Figure S6: Dependence of the properties of the antigenic clusters on the threshold D in the model with epistasis. From top to bottom the three lines of panels correspond to $D = 2$, $D = 3$ and $D = 4$, respectively. The curves for $D = 4$ are the same reported in Fig. 3 of the main text. For each value of D we report (left) the distributions of the Hamming distances h (number of homologous sites at which two strains differ) between pairs of strains assigned to the same antigenic cluster (Intra-cluster), or assigned to two consecutive clusters (Inter-clusters). The plots are averages over all the available antigenic clusters. Further the central plots report the Intra-cluster distributions for the same data, separately reported for each antigenic cluster. Finally the right plots report the Inter-clusters distributions for the same data, separately reported for each pair of consecutive antigenic clusters. The values of the parameters are set as in Fig. S3.

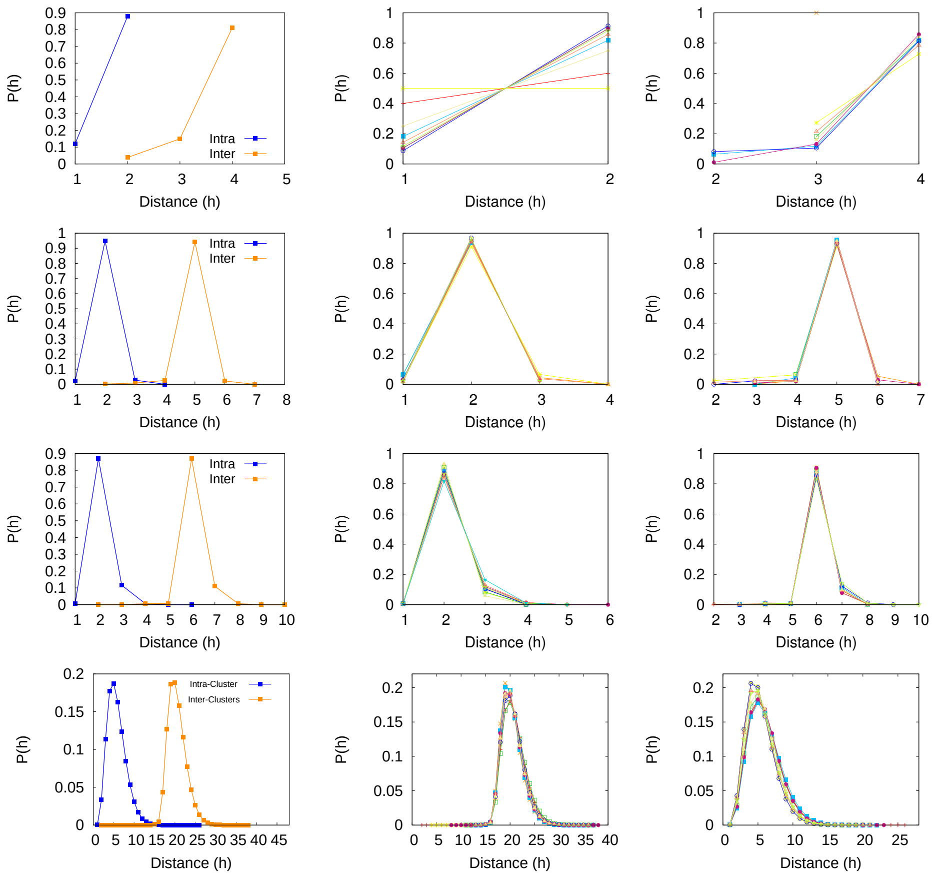

Figure S7: Properties of the antigenic clusters in the model without epistasis. From top to bottom the three lines of panels correspond to $D = 2$, $D = 3$, $D = 4$ and $D = 15$, respectively, with the same structure of Fig. S6. The curves for $D = 4$ are the same reported in Fig. 2, main text.

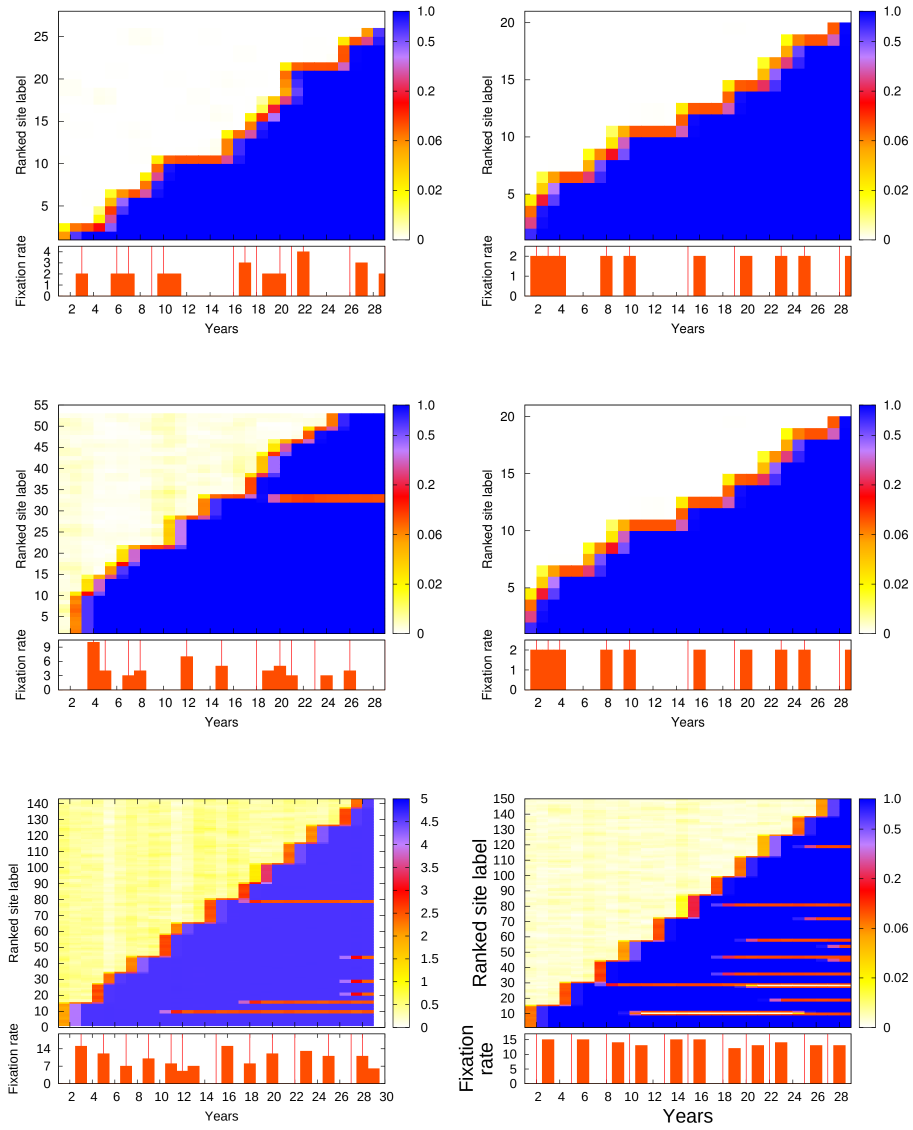

Figure S8: Patterns of fixations of nucleotide substitutions. The values of the parameters are as in Fig. S3. We consider the same observables as in Fig. 3, main text. Left column: model with epistasis. Right column: model without epistasis. First row: $D = 2$. Second row: $D = 3$. Third raw: $D = 4$ for the model with epistasis (same figure as in the main text) and $D = 15$ for the model without epistasis.

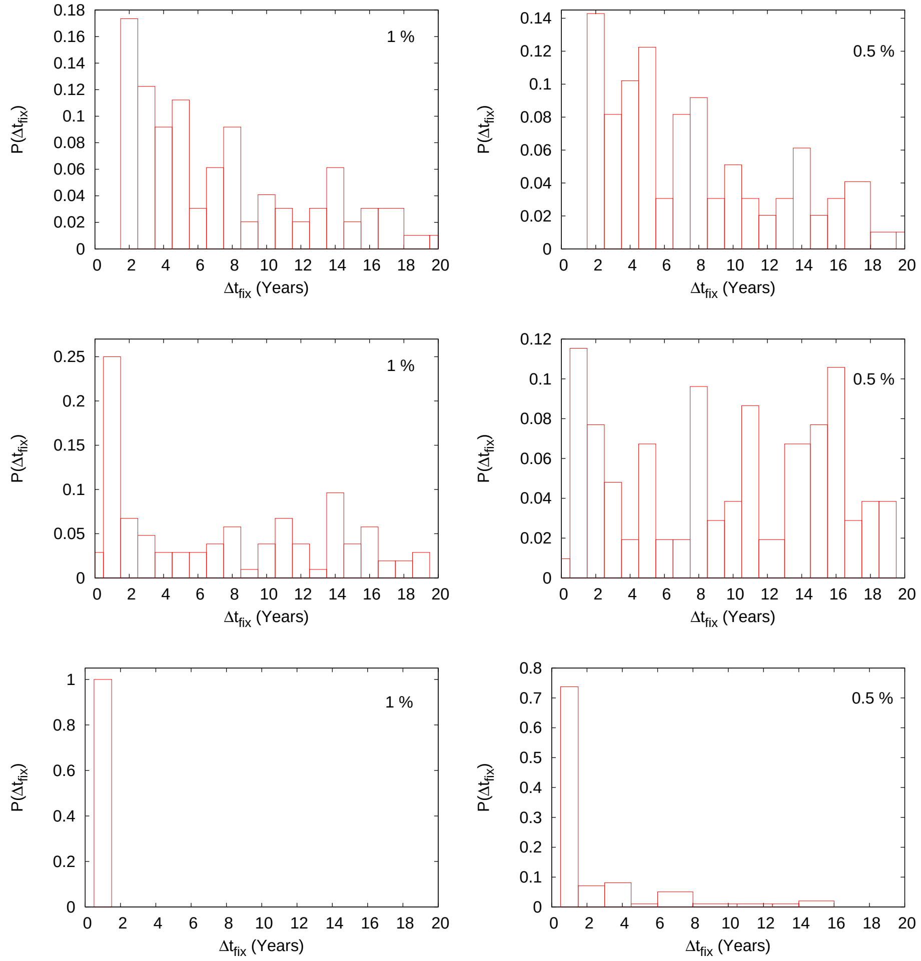

Figure S9: Histograms of the fixation times Δt_{fix} for substitutions. Here the fixation time is defined as the timespan between the first occurrence of a substitution, defined as present in at least 1% of the circulating strains (left column) or in at least 0.5% of the circulating strains (right column), to its fixation (defined as present in 95% of the circulating strains). From top to bottom: results for real data, results for the model with epistasis and $D = 4$, results for the model without epistasis and $D = 15$. The data reported in the left top and left center figures are the same as those shown in Fig. 3D in the main text.

2.2 Scaling with the population size N and with the mutation rate μ

In this section we quantify the dependence of the model predictions on the population size N as well as on the mutation rate μ. We focus in particular on the scaling laws for the three values of the antigenic threshold considered, namely $D = 2, 3, 4$, and both for the model with and without epistasis.

Let us first define two quantities we shall consider to monitor the model behavior. We first consider the effective number of antigenic clusters responsible for the infection at a given time, as measured by the *Inverse Participation Ratio* (IPR):

$$IPR(t) = \frac{(\sum_{k=0}^{N_c(t)} m_k(t))^2}{\sum_{k=0}^{N_c(t)} m_k(t)^2}, \tag{1}$$

where $N_c(t)$ is the number of antigenic clusters coexisting at time t and $m_k(t)$ the number of infections caused by the strains belonging to the cluster k at time t. We then consider the average value of the IPR over the whole evolution and infection process:

$$\langle IPR \rangle = \frac{\sum_{t=0}^{t_{max}} IPR(t)}{t_{max}}, \tag{2}$$

where t_{max} is the number of times we sample the IPR over the process.

The second observable we consider is the average time, $\Delta t_{clusters}$, elapsed between the appearance of two consecutive clusters in the population. We define the interval between the cluster k and $k+1$ as $\Delta t_{k,k+1} = t_{k+1} - t_k$, where t_k is the first time when the first strain of the cluster k infects one of the hosts in the population, and analogously t_{k+1} for the cluster $k+1$.

The quantity $\Delta t_{clusters}$ is thus defined as it follows:

$$\Delta t_{clusters} = \frac{\sum_{k=0}^{N_c} \Delta t_{k,k+1}}{N_c}, \tag{3}$$

where N_c is the total number of the clusters ever appeared in the whole process.

We now present the analysis of the scaling properties of the model, i.e., we investigate the dependence of $\langle IPR \rangle$ and $\Delta t_{clusters}$ on the size N of the system and on the mutation rate μ. We find they obey the relation $\langle IPR \rangle = f(\mu N^{\alpha(D)})$ and $\langle IPR \rangle = g(\mu N^{\alpha(D)})$, where the exponent α depends only on the threshold value D and not on the particular model (with or without epistasis) considered.

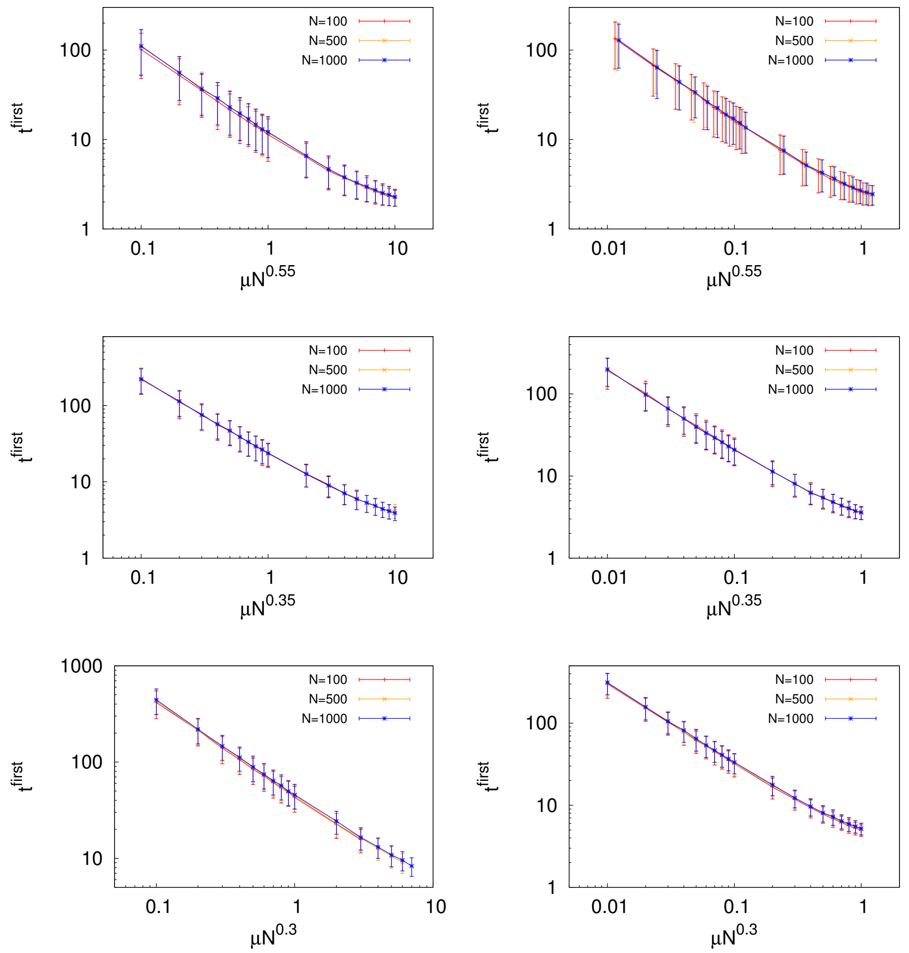

Figure S10: Average first exit time t^{first} as a function of μN^{α} in the first exit toy model considered in the text. Left column: results for the toy model where the antigenic distance is defined in terms of correlated mutations (as in the complete model with epistasis). Right column: results for the toy model where the antigenic distance is defined in terms of uncorrelated mutations (as in the complete model without epistasis). From top to bottom we report the results for $D = 2$, $D = 3$, and $D = 4$ respectively. We find that the exponents α do not depends on the definition of the antigenic distance in terms of correlated or uncorrelated mutations, but only depends on the threshold D fixed in order to escape an antigenic cluster. We find in particular: $\alpha = 0.55$ when $D = 2$, $\alpha = 0.35$ when $D = 3$ and $\alpha = 0.3$ when $D = 4$.

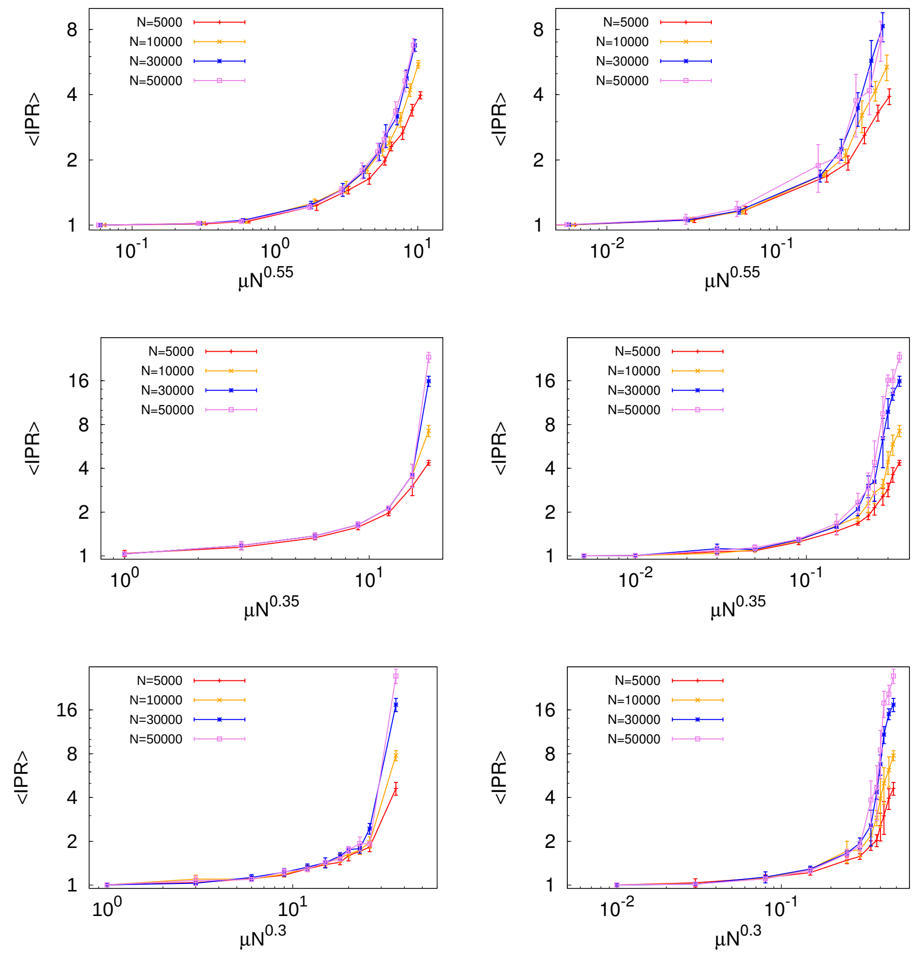

Figure S11: $\langle IPR \rangle$ as a function of μN^{α}. Curves of the Inverse Participation Ratio $\langle IPR \rangle$ as a function of the rescaled mutation rate μN^{α}. Left column: results for the model with epistasis. Right column: results for the model without epistasis. From top to bottom we report the results for $D = 2$, $D = 3$, and $D = 4$ respectively. As in Fig. S10, we find $\alpha = 0.55$ when $D = 2$, $\alpha = 0.35$ when $D = 3$ and $\alpha = 0.3$ for $D = 4$, for both the models with and without epistasis.

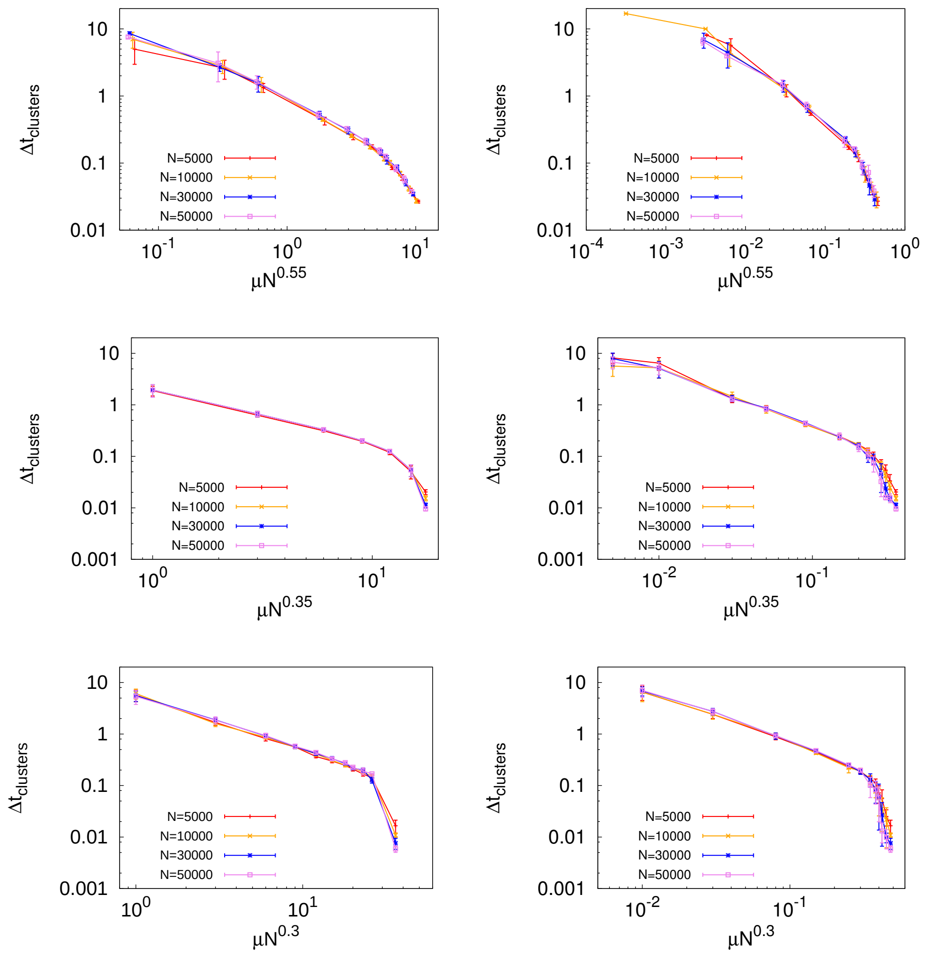

Figure S12: $\Delta t_{clusters}$ as a function of μN^{α}. Curves of the average time elapsed between the appearance of two consecutive clusters $\Delta t_{clusters}$ as a function of the rescaled mutation rate μN^{α}. Left column: results for the model with epistasis. Right column: results for the model without epistasis. From top to bottom we report the results for $D = 2$, $D = 3$, and $D = 4$ respectively. As in Fig. S10, $\alpha = 0.55$ when $D = 2$, $\alpha = 0.35$ when $D = 3$ and $\alpha = 0.3$ when $D = 4$, for both the models with and without epistasis.

It turns out that the exponent α we observe numerically can be predicted in the framework of a first exit problem. The scaling we find corresponds in fact to the dependence on μ and N of the average waiting time for the emergence of a new cluster of immunity in the following toy model. We start at time $t = 0$ with N identical binary strains that evolve at each time step with a mutation rate μ. We call t^{first} the time at which a new antigenic cluster appears (jumps in new antigenic cluster are determined as in the complete models, with or without epistasis respectively). In Fig. S10 we report the average values of t^{first}, over 1000 realizations of the toy model, as a function of $\mu N^{\alpha(D)}$, for different values of N. In all cases we obtain a scaling relation $t^{first} = t^{first}(\mu N^{\alpha(D)})$ with $\alpha = 0.55$ when $D = 2$, $\alpha = 0.35$ when $D = 3$ and $\alpha = 0.3$ when $D = 4$.

In Fig. S11 and Fig. S12 we check the scaling exponents found in the toy model in the framework of the complete models with and without epistasis. We show in particular $\langle IPR \rangle$ (Fig. S11) and $\Delta t_{clusters}$ (Fig. S12), for the complete models both with and without epistasis, again as a functions of $\mu N^{\alpha(D)}$ and for different values of N. The scaling exponents $\alpha(D)$ found in the toy model are shown to be valid also in the complete models. In particular, we observe that the collapse of the different curves is realized only for not too large values of μ ($\langle IPR \rangle \leq 5$ and $\Delta t_{clusters} > 0.05$ years, respectively), ceasing to be valid only in the not realistic regime where $\langle IPR \rangle > 5$ and $\Delta t_{clusters} < 0.05$, when there is coexistence of many antigenic clusters in the population.

In Fig. S13 we report the mean substitution rates of the strains in our model, as a function of $\Delta t_{clusters}$, the average time elapsed between the appearance of two consecutive clusters. The substitution rates were computed from the metrical distance in the phylogenetic tree of each leaf i from the $root$ (the sum of the lengths of all the branches in the path connecting i to the root). The rate at which the average metrical distance of the leaves from the root varies in time (year of sampling) defines the substitution rate. The results do not depend on the population size N, this dependence being already included in the value of $\Delta t_{clusters}$. In a realistic regime, where $\Delta t_{clusters} = 2 \div 4$ years, a realistic value for the substitution rate is observed only with $D = 4$, in the framework of our model with epistasis.

2.3 Dependence on the basic reproductive number R_0 and on the amplitude of seasonal fluctuations α.

We here investigate how the model results depend on the values of the the basic reproductive number R_0 and on the amplitude of seasonal fluctuations α. It turns out

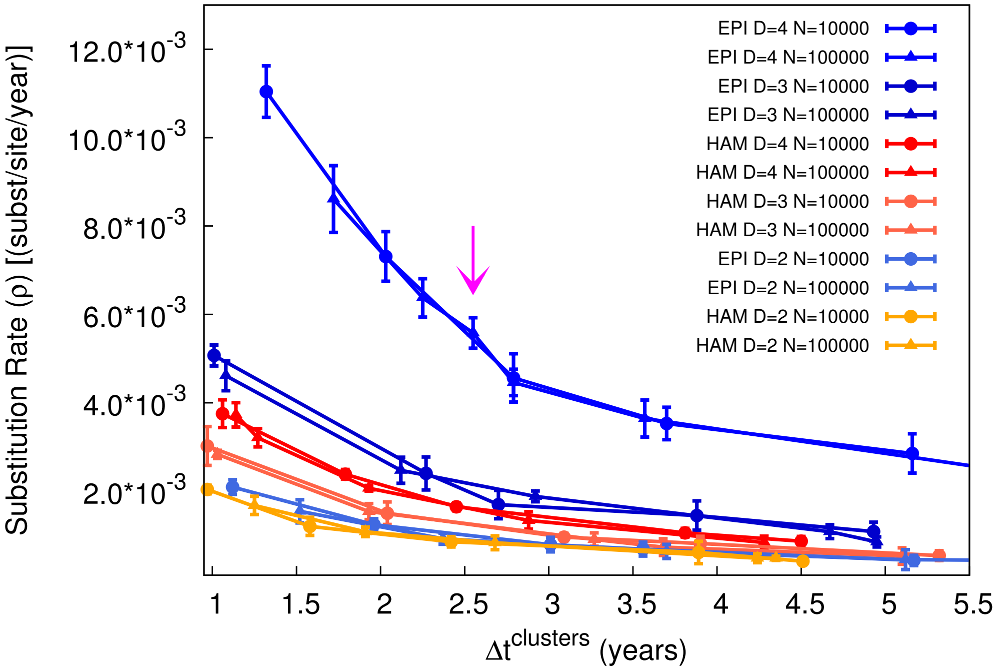

Figure S13: **Substitution Rate.** Substitution rate of the strains in the model, as a function of the average time elapsed between the appearance of two consecutive clusters $\Delta t_{clusters}$. Data shown for the two models with and without epistasis, for the values $D = 2, 3, 4$, and for two values of the population size $N = 10000, 100000$. The arrow indicates the point corresponding to the parameters values as in the main text, quantitatively reproducing the Influenza A behavior. The dependence of $\Delta t_{clusters}$ on models parameters is shown in figure S12.

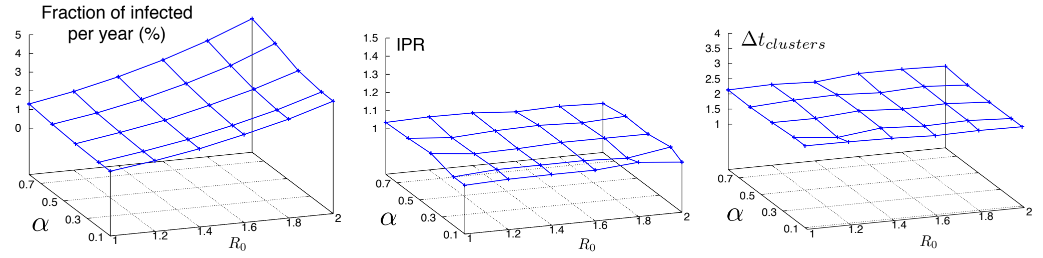

Figure S14: Dependence of the model observables on the basic reproductive number R_0 and on the amplitude of seasonal fluctuations α. Average number of infected individuals per year (Left panel), $\langle IPR \rangle$ (Central panel) and $\Delta t_{clusters}$ (Right panel) as a function of R_0 and α, for the model with epistasis and $D = 4$. All the other parameters are set as in the main text.

that these values affect only the fraction of infected individuals in the population, leaving unaltered the other properties of the model (Fig. S14). In particular, the percentage of infected individuals in the population increases when both the basic reproductive number and the amplitude of seasonal fluctuations increase (Fig. S14 left panel).

2.4 Dependence on the emigration $\gamma_{\mathcal{T}\to\mathcal{R}}$ and immigration $\gamma_{\mathcal{R}\to\mathcal{T}}$ rates.

In this section we discuss the role of the emigration and immigration rates. It turns out that these values affect only the fraction of infected individuals in the population, leaving unaltered the other properties of the model (Fig. S15). In particular, the model seems to be insensitive to the variation of the emigration rate over a wide spectrum, while the immigration rate regulates the infection level in the population (Fig. S15 left panel).

2.5 Dependence on the cross-immunity parameter σ

We investigate here the how the model results depend on the value σ, representing the cross-immunity mutually elicited against each other by two strains having antigenic distance greater than D. As shown in Fig. S16, this parameter only affects the fraction of infected individuals in the population, leaving unaltered the other properties of the model.

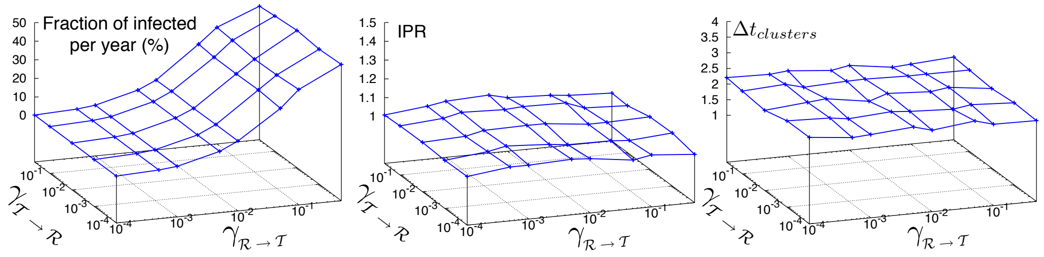

Figure S15: Dependence of the model observables on the emigration and immigration parameters. Average number of infected individuals per year (Left panel), $\langle IPR \rangle$ (Central panel) and $\Delta t_{clusters}$ (Right panel) as a function of $\gamma_{\mathcal{T}\to\mathcal{R}}$ and $\gamma_{\mathcal{R}\to\mathcal{T}}$, for the model with epistasis and $D = 4$. All the other parameters are set as in the main text.

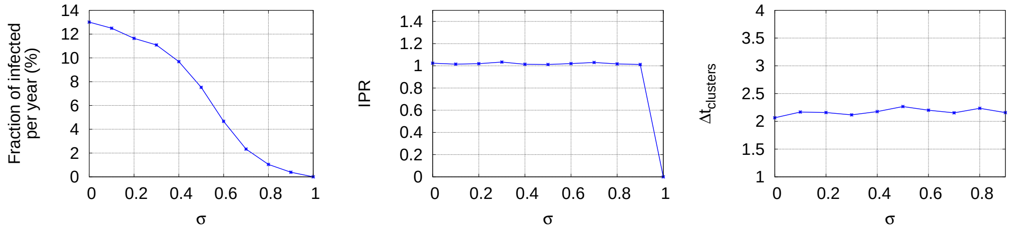

Figure S16: Dependence of the model observables on the cross-immunity parameter σ. Average number of infected individuals per year (Left panel), $\langle IPR \rangle$ (Central panel) and $\Delta t_{clusters}$ (Right panel) as a function of R_0 and α, for the model with epistasis and $D = 4$. All the other parameters are set as in the main text.

3 Influenza database

We considered 6859 strains of the H3N2 virus collected between 1988 and 2011, downloaded form GenBank [1]. The list of all the sequences considered is reported in the available file `sequence_list.txt`. For this data-set we have first performed a multiple alignment, making use of the online version of MAFFT [2], which allows for fast and accurate alignment when dealing with large data-sets, and we have then extracted the HA1 region of the haemagglutinin (HA) gene, consisting of 987 nucleotides.

3.1 Phylogenetic tree reconstruction and imbalance metric

The phylogenetic tree for the human Influenza A is inferred from the sequence database described above.

The phylogenetic tree for the models are inferred from the strains appeared in the temperate region $\mathcal{T}$. During the whole simulation, strains are collected and labelled with the year of their appearance and, for every year, a strain is sampled with a probability proportional to the total number of hosts it has infected. The total number of strains collected in each year is then a percentage (η) of the non-identical strains appeared in $\mathcal{T}$, with η varying in the range $2\% \div 6\%$.

All the phylogenetic trees are reconstructed with the distance-based algorithm Fast-SBiX [3, 4]. Pairwise distances have been computed according to the *Jukes-Cantor Model* [5, 6] in the case of the human Influenza A tree, and its equivalent model (namely the *Neyman Model* [7]) for the case of binary sequences sampled in $\mathcal{T}$.

3.1.1 Mean depth d

We here briefly recall the definition of the mean depth d [8] of a phylogenetic tree:

$$d = \frac{1}{A} \sum_{j \in (\mathcal{I} \cup \mathcal{L})} M_j, \tag{4}$$

where $\mathcal{I}$ denotes the set of the internal nodes and $\mathcal{L}$ the set of the leaves of the tree. A is the tree size defined as the total number of internal nodes and leaves of the tree: $A = 2N - 1$ in a rooted binary tree. Here M_j is the topological distance of the jth element of the tree (leaf or internal node) from the root.

In order to properly quantify the imbalance level of a phylogenetic tree, which reflects the uneven distribution of the survival ability of coexisting strains, we

make use of a sampling procedure as described in [9], that allows to perform statistical analysis on a single phylogenetic tree. We thus obtain the average value for the index d, as a function of the tree sizes $A' = 2N' - 1 \leq A = 2N - 1$. The behavior observed for the Influenza tree is of the form $\overline{d}_{flu}(A) \sim log(A)^{2.97}$ [9].

3.2 Antigenic clusters classification

Strains were assigned to a cluster of immunity according to their year of isolation. Every year the *WHO* [10] (World Health Organization) provides a vaccine composition recommendation which is based on the dominant strain circulating in the world. In Tab. S1 we report the vaccine composition recommendations for every year from 1988 to 2011, as well as the ID of the cluster of immunity, assigned according to the vaccine composition recommendation for the correspondent year.

References

[1] Benson, D. A. *et al.* Genbank. *Nucleic Acids Research* **30**, 17–20 (2002).

[2] Katoh, K., Asimenos, G. & Toh, H. Multiple alignment of dna sequences with mafft. *Methods in Molecular Biology* **537**, 39–64 (2009).

[3] Tria, F., Caglioti, E., Loreto, V. & Pompei, S. A fast noise reduction driven distance-based phylogenetic algorithm. *Proceedings of BIOCOMP2010 - The 2010 International Conference on Bioinformatics & Computational Biology* (2010).

[4] Tria, F., Caglioti, E., Loreto, V. & Pagnani, A. A stochastic local search algorithm for distance-based phylogeny reconstruction. *Molecular Biology and Evolution* **27**, 2587–2595 (2010).

[5] Jukes, T. H. & Cantor, C. R. *Evolution of Protein Molecules* (New York Academy Press, 1969).

[6] Cantor, C. R. & Jukes, T. H. The repetition of homologous sequences in the polypeptide chains of certain cytochormes and globins. *Proceedings of the National Academy of Sciences of the United States of America* **56**, 177–184 (1966).

Year of Isolation	Vaccine Composition Recommendations (source WHO)	Cluster ID
1988	A/Sichuan/02/87(H3N2)	I
1989	A/Beijing/353/89(H3N2)	II
1990	A/Beijing/353/89(H3N2)	II
1991	A/Beijing/353/89(H3N2)	II
1992	A/Beijing/32/92(H3N2)	III
1993	A/Shangdong/9/93(H3N2)	IV
1994	A/Johannesburg/33/94(H3N2)	V
1995	A/Wuhan/359/95(H3N2)	VI
1996	A/Wuhan/359/95(H3N2)	VI
1997	A/Sydney/5/97(H3N2)	VII
1998	A/Sydney/5/97(H3N2)	VII
1999	A/Moscow/10/99(H3N2)	VIII
2000	A/Moscow/10/99(H3N2)	VIII
2001	A/Moscow/10/99(H3N2)	VIII
2002	A/Fujian/411/2002(H3N2)	IX
2003	A/Fujian/411/2002(H3N2)	IX
2004	A/Wellington/1/2004(H3N2)	X
2005	A/Wisconsin/67/2005(H3N2)	XI
2006	A/Wisconsin/67/2005(H3N2)	XI
2007	A/Brisbane/10/2007(H3N2)	XII
2008	A/Brisbane/10/2007(H3N2)	XII
2009	A/Perth/16/2009(H3N2)	XIII
2010	A/Perth/16/2009(H3N2)	XIII
2011	A/Victoria/361/2011(H3N2)	XIV

Table S1: **Clusters Classification**. In this table we report the vaccine composition recommendations for every year since 1988 to 2011 [10]. In the last column we report the ID of the cluster of immunity, assigned according to the vaccine composition recommendation for the correspondent year. For example all the strains isolated in 1989, 1990 and 1991 have been assigned to the same cluster of immunity (namely cluster II), since in these years the same vaccine composition was recommended.

[7] Neyman, J. *Molecular Studies of evolution: a source of novel statistical problems* (New York Academy Press, 1971).

[8] Herrada, E. A., Eguíluz, V. M., Hernández-García, E. & Duarte, C. M. Scaling properties of protein family phylogenies. *BMC Evolutionary Biology* **11**, 155 (2011).

[9] Pompei, S., Loreto, V. & Tria, F. Phylogenetic properties of RNA viruses. *PLoS ONE* **7**, e44849 (2012).

[10] WHO recommendations on the composition of influenza virus vaccines. http://www.who.int/influenza/vaccines/virus/recommendations/en/ (2012).
